# Supplementary material for: Cost-utility analysis of Coronary Artery Calcium screening to guide statin prescription among intermediate-risk patients in Thailand
Source: PLoS One. 2025 Aug 21;20(8):e0330425. doi: 10.1371/journal.pone.0330425 (PMC12370023; doi:10.1371/journal.pone.0330425)
Supplement: S1 File — (DOCX) [file pone.0330425.s001.docx]

**Model Summary**

A hybrid model combining a decision tree and Markov model was constructed using Microsoft Excel with the Plant-A-Tree add-in [1]. Quality-adjusted life-years (QALYs) were used as the outcome utility measure. The model evaluated the target population of statin-naïve individuals aged 40-75 with intermediate cardiovascular (CV) risk, assessed using Thai CV risk tools [2], who were free of known CVD events.

1. **Epidemiological data**
   1. Incidence data on initial LDL level (mg/dl) was retrieve from Chiang Mai University Hospital database from 2012 -2022. This data pertains to intermediate-risk patients who underwent CAC testing, were free of known CVD events, and had not started statin therapy.

**Table S1**. **Patient characteristic in Hospital database**

| **Characteristic** | **n = 112**  **n(%)** |
| --- | --- |
| Male | 40 (36.0) |
| Age (years) Mean± SD | 63.4±7.62 |
| Diabetic mellitus | 15 (14.0) |
| Hypertension | 68 (63.6) |
| Chronic kidney disease | 11 (10.2) |
| Current smoker | 2 (1.9) |
| Former smoker | 5 (4.9) |
| Triglyceride, mg/dL Median (IQR) | 102 (75, 141.5) |
| LDL-C, mg/dL Median (IQR) | 106 (81, 135) |
| HDL-C, mg/dL Median (IQR) | 54 (45, 65) |
| CAC score |  |
| 0 | 28 (25.7) |
| 1- 99 | 34 (31.2) |
| ≥100 | 47 (43.1) |
|  |  |

**Abbreviation:** IQR, interquartile range; LDL, low density lipoprotein; SD, standard deviation

**1.2** Transitional probabilities in “No CVD” health state were based on the incidence of CVD events from Tainsuwan et al. [3]. The cohort data was sourced from the Advanced Diagnostic Imaging Center at Ramathibodi Hospital, Mahidol University, covering the period from November 2005 to November 2013. The composite outcome of interest was encompassed of CV death, non-fatal MI, and non-fatal stroke

**Table S2. Baseline characteristics of Tainsuwan cohort.**

| Baseline Characteristic | Intermediate-risk (10-<20%) n=1427 |
| --- | --- |
| CAC score level, n (%) |  |
| 0 | 370 (25.9) |
| 1-99 | 551 (38.6) |
| >100 | 506 (35.5) |
| Ln(CAC +1), median (range) | 3.7 (0.0, 8.1) |
| CAVI |  |
| ≥ 9 | 855 (60.6) |
| < 9 | 555 (39.4) |
| Age, years, mean (SD) | 66.22 (5.9) |
| Sex, n (%) |  |
| Male | 714 (50.0) |
| Female | 713 (50.0) |
| BMI, kg/m^2^, mean (SD) | 25.53 (3.6) |
| DM, n (%) |  |
| Yes | 734 (51.4) |
| No | 693 (48.6) |
| Hypertension, n (%) |  |
| Yes | 1247 (87.4) |
| No | 180 (12.6) |
| SBP, mmHg, mean (SD) | 142.65 (15.8) |
| Dyslipidemia, n (%) |  |
| Yes | 1011 (70.9) |
| No | 416 (29.2) |
| Current/ex-Smoking, n (%) |  |
| Yes | 340 (23.8) |
| No | 1087 (76.2) |
| CKD, n (%) |  |
| Yes | 163 (11.4) |
| No | 1264 (88.6) |
| Uric acid >7 mg/dL |  |
| Yes | 262 (18.4) |
| No | 1161 (81.6) |
| Family history of CAD, n (%) |  |
| Yes | 385 (27.0) |
| No | 1042 (73.0) |
| TC, mg/dL, mean (SD) | 201.41 (40.5) |
| HDL-C, mg/dL, mean (SD) | 47.86 (11.6) |
| LDL-C, mg/dL, mean (SD) | 127.28 (36.8) |
| Statins, n (%) |  |
| Yes | 1127 (79.0) |
| No | 300 (21.0) |
| Anti-hypertensive agents, n (%) |  |
| Yes | 1137 (79.7) |
| No | 290 (20.3) |

**This table was modified from the original table.** **Abbreviation:** BMI, body mass index; CAD, coronary artery disease; CAVI, cardio-ankle vascular index; CKD, chronic kidney disease (eGFR < 60 ml/min/1.73 ml^2^); DM, diabetes mellitus; HDL-C, high-density lipoprotein cholesterol; LDL-C, low density lipoprotein cholesterol; SBP = systolic blood pressure; TC = total cholesterol

**1.3** CAC progression every five years was estimated based on data from the Chiang Mai University Hospital database (2012-2022). This data was fitted using ordinal logistic

regression, adjusting for age, sex, and co-morbidities (hypertension, diabetes, and chronic kidney disease).

**Figure S2. Probability of CAC progression by age**

**
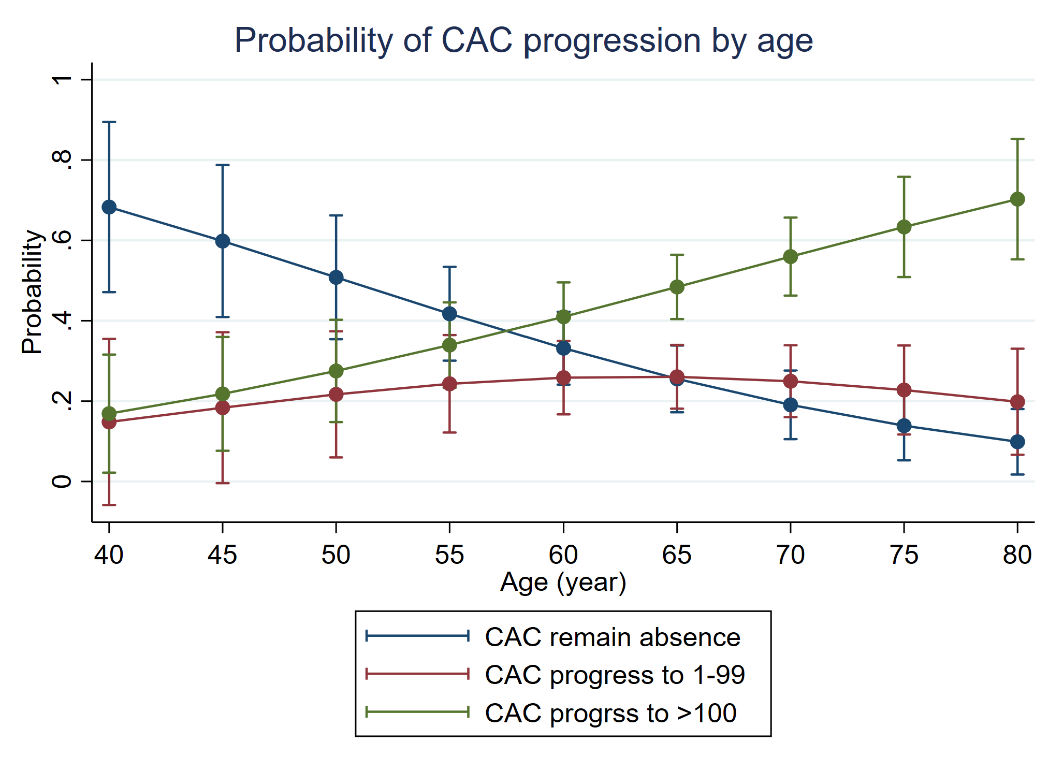
**

To explore the impact of CAC progression over age, we conducted scenario analysis using CAC progression data estimated from a prediction model based on the CAC Consortium [7], a multicenter cohort study involving four high-volume centers in the United States (Total N= 22,346 patients)

**Table S3. Comparison of the probability of CAC progression over age among patient who initially have no detectable CAC**

| **Baseline characteristic** | **Hospital data based** 2012-2022 | | **CAC Consortium [4]** |
| --- | --- | --- | --- |
|  | Age (years) Mean± SD: 63.4±7.62  Intermediate risk: 100%  CAC score categories (%): 0 (25.7), 1-99 (31.2), ≥100 (43.1) | | Age (years) Mean± SD: 43.5±4.5  Intermediate risk: 2.6%  CAC score categories (%): 0 (65.6), 1-99 (27.2), ≥100 (7.2) |
| Age | Probability of having CAC  1-99, ≥ 100 | Probability of having CAC  ≥ 0 | Probability of having CAC  ≥ 0 |
| 45 – 49 | 0.341, 0.158 | 0.50 | 0.17 |
| 50 – 54 | 0.338, 0.195 | 0.53 | 0.26 |
| 55 – 59 | 0.331, 0.237 | 0.57 | 0.38 |
| 60 – 64 | 0.319, 0.284 | 0.60 | 0.50 |
| 65 – 69 | 0.302, 0.336 | 0.64 | 0.63 |
| 70 – 74 | 0.281, 0.390 | 0.67 | 0.74 |

**Abbreviation: CAC, coronary artery calcium; SD, standard deviation**

**Table S4. non-CV death, utility of Thailand population by age**

| age |  | Death (Gen pop) | Utility gen pop |
| --- | --- | --- | --- |
|  |  |  |  |
| 40 |  | 0.0038 | 0.941 |
| 41 |  | 0.0038 | 0.941 |
| 42 |  | 0.0038 | 0.941 |
| 43 |  | 0.0038 | 0.941 |
| 44 |  | 0.0038 | 0.941 |
| 45 |  | 0.0050 | 0.923 |
| 46 |  | 0.0050 | 0.923 |
| 47 |  | 0.0050 | 0.923 |
| 48 |  | 0.0050 | 0.923 |
| 49 |  | 0.0050 | 0.923 |
| 50 |  | 0.0069 | 0.923 |
| 51 |  | 0.0069 | 0.923 |
| 52 |  | 0.0069 | 0.923 |
| 53 |  | 0.0069 | 0.923 |
| 54 |  | 0.0069 | 0.923 |
| 55 |  | 0.0099 | 0.886 |
| 56 |  | 0.0099 | 0.886 |
| 57 |  | 0.0099 | 0.886 |
| 58 |  | 0.0099 | 0.886 |
| 59 |  | 0.0099 | 0.886 |
| 60 |  | 0.0141 | 0.886 |
| 61 |  | 0.0141 | 0.886 |
| 62 |  | 0.0141 | 0.886 |
| 63 |  | 0.0141 | 0.886 |
| 64 |  | 0.0141 | 0.886 |
| 65 |  | 0.0211 | 0.862 |
| 66 |  | 0.0211 | 0.862 |
| 67 |  | 0.0211 | 0.862 |
| 68 |  | 0.0211 | 0.862 |
| 69 |  | 0.0211 | 0.862 |
| 70 |  | 0.0320 | 0.862 |
| 71 |  | 0.0320 | 0.862 |
| 72 |  | 0.0320 | 0.862 |
| 73 |  | 0.0320 | 0.862 |
| 74 |  | 0.0320 | 0.862 |
| 75 |  | 0.0503 | 0.862 |

1. **Cost data**
   1. **Statin cost and distribution**

**Table S5. Estimated annual statin cost based on hospital database dispensing distribution.**

| **Statin intensity** | **Statin** | **Daily dose** | **Unit Cost, 2024 Baht** | **Proportion^*^** | **Average unit cost, Baht** | **Average annual cost, Baht** | **ICER** |
| --- | --- | --- | --- | --- | --- | --- | --- |
| moderate | Atorvastatin | 20mg | 15.15 | 40.9% | 7.09 | 2589.091 | 21,813.6 |
|  | Simvastatin | 40mg | 1.5 | 51.1% |  |  |  |
| High | Atorvastatin | 40mg | 25 | 95.4% | 24.83 | 9071.366 |  |
|  | Rosuvastatin | 20mg | 21.4 | 4.6% |  |  |  |

*prescribing proportions data was retrieved the hospital database from 2021 to 2024

**2.2 Details of study selection for CVD event cost parameters**

**Table S6. Source of cost data referenced by previous studies**

| **Study** | **Up front / first year MI cost** | **Annually post MI cost** | **Up front/ first year stroke cost** | **Annually stroke cost** |
| --- | --- | --- | --- | --- |
| Khonputsa et.al. 2012 [4] | 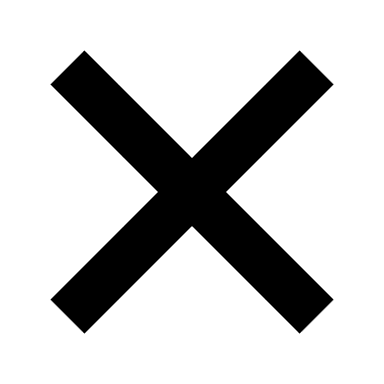 | 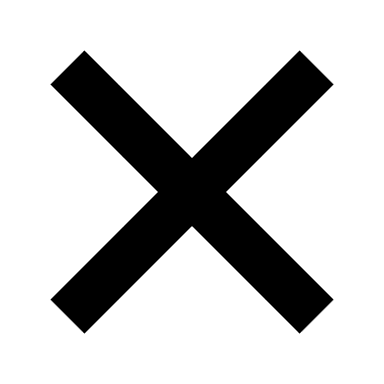 | 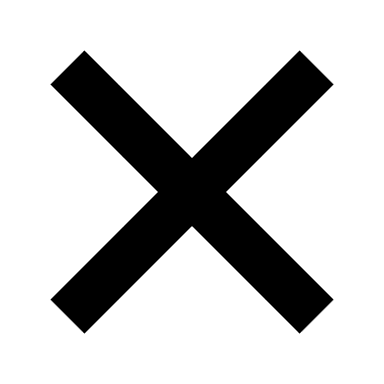 | 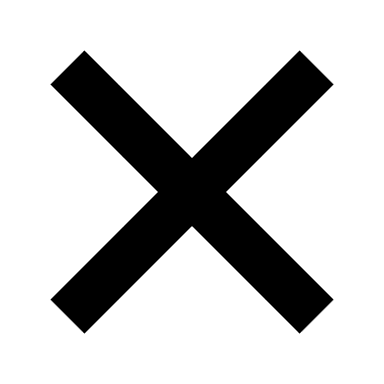 |
| S. Jarungsuccess et.al. 2014 [5] | Admission cost per 1 event  93,882  (75,380-112,384) | 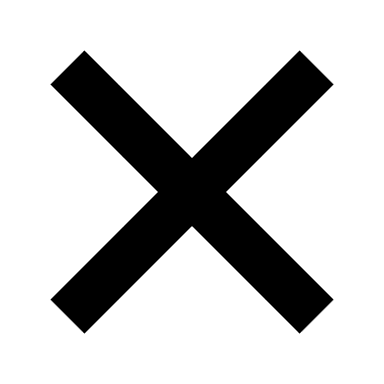 | Admission cost per 1 event 24,604  (21,686-27,522) | 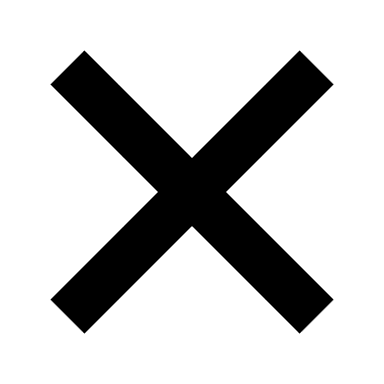 |
| Kongpakwattana et.al. 2019 [6] | referring to Anukoolsawat et al. (2006) [9] | referring to Anukoolsawat et al. (2006) [9] | referring to Tamteeranon eat.al 2008 [10] | referring to Tamteeranon eat.al 2008 [10] |
| Borttiger et.al. 2021 [7] | referring to Anukoolsawat et al. (2006) [9] | referring to Anukoolsawat et al. (2006) [9] | referring to Tamteerano eat.al 2008 [10] | referring to Tamteerano eat.al 2008 [10] |
| Rattanavipapong et.al.2022 [8] |  |  | derived from Siriraj Stroke Center from 2015 to 2021 | derived from Siriraj Stroke Center from 2015 to 2021 |

**Details of Cost Parameters from Anukoolsawat et al. (2006) and Rattanavipapong et.al. (2022)**

Our study derived costs for secondary treatment and prevention in populations who experienced non-fatal MI costs, fatal MI costs, and direct non-medical costs from Anukoolsawat et al. (2006) [9]. This study used data from the Thai Acute Coronary Syndrome (ACS) registry to estimate lifetime costs of ACS. Medical records of 330 ACS patients were used to calculate direct medical costs (pre-event and MI-related costs of non-fatal MI), while interview records of 193 ACS patients were used to estimate direct non-medical costs. For the costs of non-fatal stroke and recurrent non-fatal stroke (both direct and indirect), our study utilized data from Rattanavipapong et al. (2022) [8] due to its recency and comprehensive. This study utilized retrospective patient data from a multicentre stroke unit, incorporating key parameters such as inpatient length of stay, number of follow-up visits, and travel distance to estimate non-medical costs. Furthermore, transportation, caregiver, food, and accommodation expenses incurred during treatment were referenced from Singhpoo K et al. (2009) [11]. Detailed information on direct and non-medical costs and their components can be found in the supplementary materials of the original article.

**Reference**

1. Adeagbo CU, Rattanavipapong W, Guinness L, et al. The development of the guide to economic analysis and research (GEAR) online resource for low- and middle-income countries' health economics practitioners: a commentary. Value Health2018;21:569–72
2. Prin Vathesatogkit, Mark Woodward, Supachai Tanomsup, Wipa Ratanachaiwong, Somlak Vanavanan, Sukit Yamwong, Piyamitr Sritara, Cohort Profile: The electricity generating authority of Thailand study, International Journal of Epidemiology, Volume 41, Issue 2, April 2012, Pages 359–365, https://doi.org/10.1093/ije/dyq218
3. Tiansuwan N, Sasiprapha T, Jongjirasiri S, Unwanatham N, Thakkinstian A, Laothamatas J, Limpijankit T. Utility of coronary artery calcium in refining 10-year ASCVD risk prediction using a Thai CV risk score. Front Cardiovasc Med. 2023 Nov 2;10:1264640. doi: 10.3389/fcvm.2023.1264640. PMID: 38028497; PMCID: PMC10652894.
4. Khonputsa P, Veerman LJ, Bertram M, Lim SS, Chaiyakunnaphruk N, Vos T. Generalized Cost-Effectiveness Analysis of Pharmaceutical Interventions for Primary Prevention of Cardiovascular Disease in Thailand. Value in health regional issues. 2012;1(1):15-22.
5. Jarungsuccess S, Taerakun S. Cost-utility analysis of oral anticoagulants for nonvalvular atrial fibrillation patients at the police general hospital, Bangkok, Thailand. Clinical therapeutics. 2014;36(10):1389-1394.e1384.
6. Kongpakwattana K, Ademi Z, Chaiyasothi T, Nathisuwan S, Zomer E, Liew D, Chaiyakunapruk N. Cost-Effectiveness Analysis of Non-Statin Lipid-Modifying Agents for Secondary Cardiovascular Disease Prevention Among Statin-Treated Patients in Thailand. Pharmacoeconomics. 2019 Oct;37(10):1277-1286. doi: 10.1007/s40273-019-00820-6. PMID: 31243736.
7. Boettiger DC, Chattranukulchai P, Avihingsanon A, Chaiwarith R, Khusuwan S, Law MG, Ross J, Kiertiburanakul S. Atherosclerotic cardiovascular disease thresholds for statin initiation among people living with HIV in Thailand: A cost-effectiveness analysis. PLoS One. 2021 Sep 9;16(9):e0256926. doi: 10.1371/journal.pone.0256926. PMID: 34499685; PMCID: PMC8428548.
8. Rattanavipapong W, Worakijthamrongchai T, Soboon B, et al. Economic evaluation of endovascular treatment for acute ischaemic stroke in Thailand. BMJ Open2022;12:e064403. doi:10.1136/bmjopen-2022-064403
9. Anukoolsawat, Pongchai, Piyamitr Sritara and Yot Teerawattananon. “Costs of Lifetime Treatment of Acute Coronary Syndrome at Ramathibodi Hospital.” (2006).
10. Tamteeranon Y, Khonputsa P, U. C, Teerawattananon Y, Lim S. *Economic evaluationof HMG-CoA reductase inhibitor (statin) for primary prevention of cardiovascular diseases among Thai population.* Bangkok2008.
11. Singhpoo K, Tiamkao S, Ariyanuchitkul S, Sangpongsanon S, Kamsa-ard S, Lekbunyasin O, Soommart Y. The expenditures of stroke outpatients at Srinagarind hospital. Srinagarind Medical Journal. 2009;24(1):54-9.
