# Supplementary figures and images for: Cost-utility analysis of Coronary Artery Calcium screening to guide statin prescription among intermediate-risk patients in Thailand
Source: PLoS One. 2025 Aug 21;20(8):e0330425. doi: 10.1371/journal.pone.0330425 (PMC12370023; doi:10.1371/journal.pone.0330425)

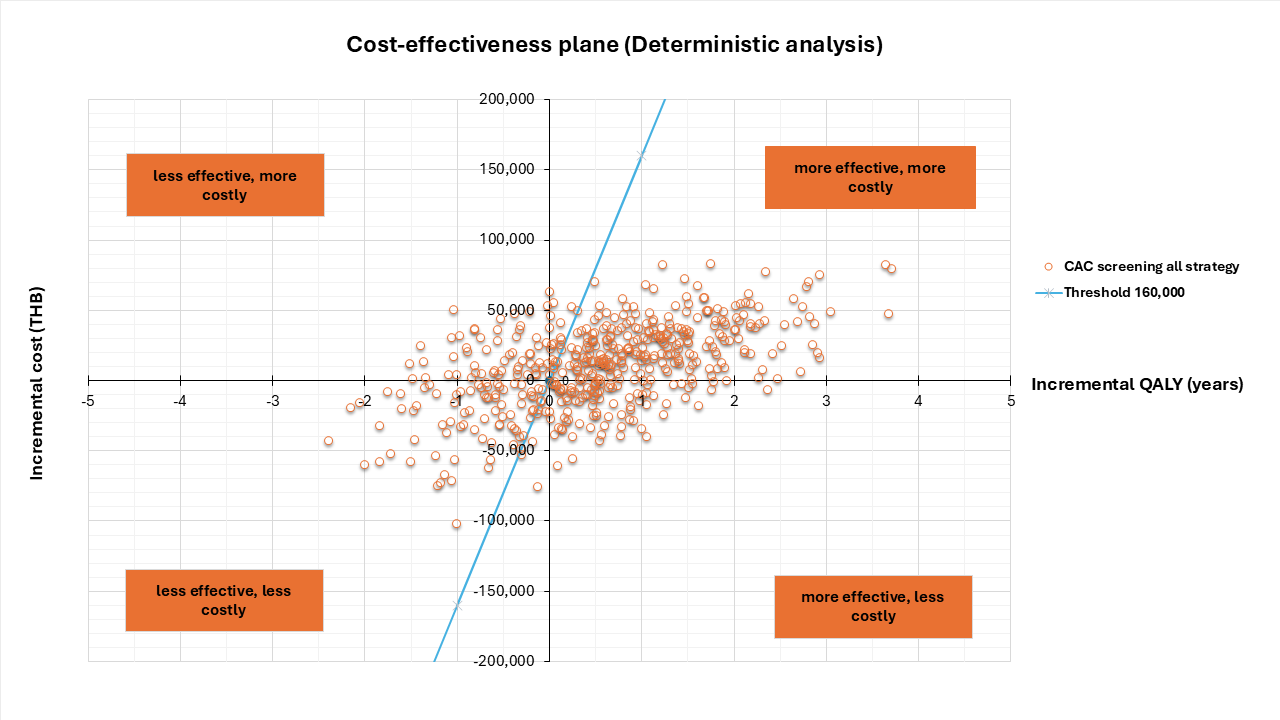

Supplement: S1 Fig — (PNG) [file pone.0330425.s002.png]
