# Supplementary material for: Cost-utility analysis of Coronary Artery Calcium screening to guide statin prescription among intermediate-risk patients in Thailand
Source: PLoS One. 2025 Aug 21;20(8):e0330425. doi: 10.1371/journal.pone.0330425 (PMC12370023; doi:10.1371/journal.pone.0330425)
Supplement: Attachment — Submitted filename: response letter 20Oct24_SS.docx [file pone.0330425.s004.docx]

**Reviewers comment on the manuscript**

**Entitles: “Cost-utility analysis of Coronary Artery Calcium screening to guide statin prescription among intermediate risk patient in Thailand”**

Dear Editor and reviewers,

We would like to thank you for your valuable reviews and comments. It is our great pleasure to have an opportunity to revise our manuscript. We have revised and modified our manuscript with some additional information (track change) as suggested by reviewers’ comment. We hope that our revisions will improve the quality of the manuscript and give a clearer vision of research methodology to meet qualification for publication in PLOS ONE. Please inform us if further information or clarification needs to be addressed. We are looking forward to your reviews and would be extremely grateful to make our response.

____________________________________________________________________________

**Reviewer #1:** The authors aim to evaluate the cost-effectiveness of using Coronary Artery Calcium (CAC) screening as an alternative to the current screening guidelines in Thailand. However, I recommend that this study be rejected for publication in PLOS ONE.
The primary concern is that the relative effect between CAC screening and the current guidelines has not been appropriately estimated. There are two key issues:

1. Initial Incidence Data: The initial incidence of LDL-C and CAC levels is derived from only 112 patients at a single hospital. This small, non-representative sample cannot adequately reflect the Thai population. Additionally, the inclusion of patients who must have CAC testing introduces selection bias.

**Answer:** We appreciated your feed back and would like to address your concern. We acknowledge that our data may not fully represent the true population of our country. However, the included patients were rigorously screened to ensure they were at intermediate cardiovascular risk and had never received statin therapy. We specifically selected patients who underwent CAC screening because our goal was to assess LDL distribution in individuals for whom CAC might be used to guide statin therapy.

We have provided incidence data for LDL categories based on previous evidence that closely aligns with our target patient population.

Table 1.

| Study | Country | ASCVD 10-year risk | Incidence distribution | Inclusion criteria | Disadvantage |
| --- | --- | --- | --- | --- | --- |
| Kevin E Kip  2024 [1] | USA | Intermediate | Total N =32,801  LDL < 70 = 8.8 %  LDL 70-189 = 88.6 %  LDL >= 190 = 2.6% | patients aged 50–89 years from tertiary care  and was restricted to primary prevention  (not on statin therapy at baseline or within 1year of follow-up.) | Other country |
| Matthew E Gold  2020 [2] | USA | Mixed | Total N =4,623,851  LDL >= 190 = 2.9% | included adults (age ≥18 years) with LDL-C ≥190 mg/dL, at least one LDL-C level drawn from 255 health systems participatin | Other country  Mixed ASCVD risk  Mixed population of patient receive statin |
| Tainsuwan N2023 [3] | Thai | intermediate | Total N= 1,427  LDL Mean ±SD  127.28 ±(36.8) | Patient underwent CAC without previous MACE event | Mixed population of patient receive statin |
| Aekplakorn W  2009 [4] | Thai | Mixed | Total N= 19,021  LDL Mean (SE)  128.7 (1.1) | NHES 2009 | Mixed population of patient receive statin |
| This study | Thai | intermediate | Total N= 112  LDL median (IQR)  106 (81, 135)  LDL < 70 = 22.8 %  LDL 70-189 = 75.9 %  LDL >= 190 = 1.8% | Chiang Mai University Hospital database from 2012 -2022  Patients were free of CVD, and not started statin therapy. | Small sample size |

Our data might be the best available data that align with our target population at the current time. The true LDL-C levels of population could be higher or lower than the values used in the base case. Sensitivity analyses were then conducted to account for uncertainties in these parameters. In the one-way sensitivity analysis, the incidence of initial LDL levels at 70 and 70-189 mg/dL showed minimal impact on the ICER, while LDL levels above 190 mg/dL emerged as a sensitive input parameter. We varied the probability of this incidence between 0% and 5% (the upper or lower limit were based on standard error), and the ICER demonstrated a trend toward negative values as the incidence increased (see red box in Figure 1). Importantly, the ICER did not exceed the willingness-to-pay (WTP) threshold of 160,000 THB when 0 was input, indicating that the conclusion for this parameter remains robust. However, we encourage the use of national data that specifically focus on collecting these input parameters in future budget impact analyses before implementation.

We have added the text regarding to this limitation in line 341-344 as follow: “Fourth, some input parameters were derived from data specific to our center with relatively have small sample size, which may not be fully representative of the Thai population. However, sensitivity analysis accounted for this parameter uncertainties showed robust outcome. We recommend rigorously collecting large national datasets for these parameters in future budget impact analyses before implementation.”

Figure 1. Tornado diagram of one-way sensitivity analysis

Reference

1. Kip KE, Diamond D, Mulukutla S, et al. Is LDL cholesterol associated with long-term mortality among primary prevention adults? A retrospective cohort study from a large healthcare system. BMJ Open2024;14:e077949. doi:10.1136/bmjopen-2023-077949
2. Gold, M. E., Nanna, M. G., Doerfler, S. M., Schibler, T., Wojdyla, D., Peterson, E. D., & Navar, A. M. (2020). Prevalence, treatment, and control of severe hyperlipidemia. *American journal of preventive cardiology*, *3*, 100079. <https://doi.org/10.1016/j.ajpc.2020.100079>
3. Tiansuwan, N., Sasiprapha, T., Jongjirasiri, S., Unwanatham, N., Thakkinstian, A., Laothamatas, J., & Limpijankit, T. (2023). Utility of coronary artery calcium in refining 10-year ASCVD risk prediction using a Thai CV risk score. Frontiers in cardiovascular medicine, 10, 1264640. https://doi.org/10.3389/fcvm.2023.1264640
4. Aekplakorn W, Taneepanichskul S, Kessomboon P, et al. Prevalence of Dyslipidemia and Management in the Thai Population, National Health Examination Survey IV, 2009. J Lipids. 2014;2014:249584. doi:10.1155/2014/249584

2. Long-Term Effect Estimation: The long-term effect of CAC screening is based on the estimated progression of CAC by age, derived from a retrospective study without a direct comparator. The progression data for comparator is sourced from another trial, making the relative effect unreliable.

**Answer:** We appreciated your feedback and would like to address your concern. Our model evaluated the impact of statin initiation for primary prevention based on the 2019 ACC/AHA guideline with CAC screening compared to without CAC screening over a 35-year time horizon. CAC progression is an input parameter used to predict the likelihood of progression to CAC 1-99 or >100 in patients who initially have no detectable CAC. This parameter was not addressed in previous models [1-5], which assumed the CAC score remained constant over time. To account for the natural progression of the disease, we included this parameter in the model to construct a more realistic cost-effectiveness analysis and to ensure fairness for patients who initially have no detectable CAC.

The observational studies that assess long-term CAC progression in Thailand is limited, as these types of studies require considerable time and resources. Estimating CAC progression every five years using retrospective data from the Chiang Mai University Hospital database (2012-2022, N=112) may be the most relevant evidence available for our target population.

The efficacy of moderate- or high-potency statins for reduction the risk of major cardiovascular events is based on an average pooled effect from meta-analysis of multiple trials, with a median follow-up of up to 5 years [6]. As a result, we assumed that the long-term effect of statin therapy remains constant across all age groups.

Additionally, we conducted scenario analysis using CAC progression data estimated from a prediction model based on the CAC Consortium [7], a multicenter cohort study involving four high-volume centers in the United States (Total N= 22,346 patients) (Table 2), and a scenario where CAC progression parameters were not used in the model. The results showed that slower CAC progression makes the CAC screening strategy more cost-effective, as fewer patients developed CAC 1-99, and ≥ 100 (Table 3). However, the probability estimates from the CAC Consortium may not be proper to be use in base-case, as they are based on a population not closely aligned with our target group.

We have added this analysis in our appendix and addressed this issue as our strength as follow:

Method section: in line 207-210

“Third, we explored the impact of CAC progression by changing the CAC progression probability using prediction model from the CAC Consortium, a large multicenter cohort of low CV patients in the USA [45] (details on the probabilities by age are provided in Supplementary Table S3).”

Result section: in line 263-265

“The results showed that slower CAC progression makes the CAC screening strategy more cost-effective, as fewer patients developed CAC 1-99, and ≥ 100 (Table 4).”

Discussion section: in line 320-325

“It was previously revealed that many patients who had non-fatal MI or stroke went on to experience other major hard events within a year [27, 28]. In the real world, the transition probabilities, utility, and costs within post non-fatal MI & stroke probably differ from other health states. Additionally, CAC scores naturally progress with age. Our study's strength lies in incorporating these health states and the CAC progression parameter into the Markov model, ensuring a more realistic representation of patient outcomes.”

**Table 2. Comparison of the probability of CAC progression over age among patient who initially have no detectable CAC**

| **Baseline characteristic** | **Hospital data based** 2012-2022 | | **CAC Consortium [7]** |
| --- | --- | --- | --- |
|  | Age (years) Mean± SD: 63.4±7.62  Intermediate risk: 100%  CAC score categories (%): 0 (25.7), 1-99 (31.2), ≥100 (43.1) | | Age (years) Mean± SD: 43.5±4.5  Intermediate risk: 2.6%  CAC score categories (%): 0 (65.6), 1-99 (27.2), ≥100 (7.2) |
| Age | Probability of having CAC  1-99, ≥ 100 | Probability of having CAC  ≥ 0 | Probability of having CAC  ≥ 0 |
| 45 – 49 | 0.341, 0.158 | 0.50 | 0.17 |
| 50 – 54 | 0.338, 0.195 | 0.53 | 0.26 |
| 55 – 59 | 0.331, 0.237 | 0.57 | 0.38 |
| 60 – 64 | 0.319, 0.284 | 0.60 | 0.50 |
| 65 – 69 | 0.302, 0.336 | 0.64 | 0.63 |
| 70 – 74 | 0.281, 0.390 | 0.67 | 0.74 |

| **Scenario** |  | **Incremental**  **Cost (THB)** | **Incremental QALYs (years)** | **ICER** | **Decision*** |
| --- | --- | --- | --- | --- | --- |
| **CAC progression parameter** | | | | | |
| Absence | | -6,866 | 0.62 | -11,014 | CAC is dominant |
| Based on CAC Consortium [7] | | 13 | 0.64 | 20 | CAC is cost-effective |
| Based on Hospital data |  | 10,091 | 0.62 | 16,308 | CAC is cost-effective |
|  |  |  |  |  |  |

**Table 3. Scenario analysis**

*Cost-effectiveness was interpreted under the Thai willingness-to-pay threshold of 160,000 THB per QALY gained. Approximately 36 THB = 1 US$ in 2024. **Abbreviation:** CAC, coronary calcium score; ICER, incremental cost-effectiveness ratio; QALYs, Quality adjusted life years.

Reference

1. van Kempen BJ, Spronk S, Koller MT, et al. Comparative effectiveness and cost-effectiveness of computed tomography screening for coronary artery calcium in asymptomatic individuals. J Am Coll Cardiol 2011;58:1690–701.
2. Roberts ET, Horne A, Martin SS, et al. Cost-effectiveness of coronary artery calcium testing for coronary heart and cardiovascular disease risk prediction to guide statin allocation: the Multi-Ethnic Study of Atherosclerosis (MESA). PLoS One 2015;10:e0116377.
3. van Kempen BJ, Ferket BS, Steyerberg EW, Max W, Myriam Hunink MG, Fleischmann KE. Comparing the cost-effectiveness of four novel risk markers for screening asymptomatic individuals to prevent cardiovascular disease (CVD) in the US population. Int J Cardiol 2016;203: 422–31.
4. Hong JC, Blankstein R, Shaw LJ, Padula WV, Arrieta A, Fialkow JA, Blumenthal RS, Blaha MJ, Krumholz HM, Nasir K. Implications of Coronary Artery Calcium Testing for Treatment Decisions Among Statin Candidates According to the ACC/AHA Cholesterol Management Guidelines: A Cost-Effectiveness Analysis. JACC Cardiovasc Imaging. 2017 Aug;10(8):938-952. doi: 10.1016/j.jcmg.2017.04.014. PMID: 28797417.
5. Galper BZ, Wang YC, Einstein AJ. Strategies for primary prevention of coronary heart disease based on risk stratification by the ACC/ AHA lipid guidelines, ATP III guidelines, coronary calcium scoring, and C-reactive protein, and a global treat-all strategy: a comparativeeffectiveness modeling study. PLoS One 2015; 10:e0138092.
6. Cholesterol Treatment Trialists' (CTT) Collaborators, Mihaylova, B., Emberson, J., Blackwell, L., Keech, A., Simes, J., Barnes, E. H., Voysey, M., Gray, A., Collins, R., & Baigent, C. (2012). The effects of lowering LDL cholesterol with statin therapy in people at low risk of vascular disease: meta-analysis of individual data from 27 randomised trials. Lancet (London, England), 380(9841), 581–590. https://doi.org/10.1016/S0140-6736(12)60367-5
7. Dzaye, O., Razavi, A. C., Dardari, Z. A., Shaw, L. J., Berman, D. S., Budoff, M. J., Miedema, M. D., Nasir, K., Rozanski, A., Rumberger, J. A., Orringer, C. E., Smith, S. C., Jr, Blankstein, R., Whelton, S. P., Mortensen, M. B., & Blaha, M. J. (2021). Modeling the Recommended Age for Initiating Coronary Artery Calcium Testing Among At-Risk Young Adults. *Journal of the American College of Cardiology*, *78*(16), 1573–1583. https://doi.org/10.1016/j.jacc.2021.08.019

**Reviewer #2:** Thank you for the thoughtful research. Here are some specific and general comments:

**Abstract:**

1. The statement in the methods “Different potencies of statin were initiated based on CAC score and ACC/AHA guidelines 2019 recommendation” is not clear to me. From the introduction my understanding is that this was comparing CAC screening plus treatment to standard treatment as directed by guidelines. Without reading the body of the paper, it’s confusing how many different interventions are being compared.

**Answer**: In our study, statin therapy was initiated at either high or moderate potency based on the severity of each individual’s cardiovascular risk. Recent studies show that the CAC score improves cardiovascular risk assessment [1], helping to better identify patients needing intensive statin therapy or those who may not benefit. Our aim is to compare the cost-effectiveness of incorporating the CAC score as a cardiovascular risk assessment tool, based on the 2019 ACC/AHA guidelines, against not using it for the primary prevention of ASCVD. For the CAC screening strategy, individual patients are assessed based on their CAC score to guide statin therapy. A moderate-potency statin is prescribed for patients with a CAC score of 1-99, while a high-potency statin is recommended for those with a CAC score of ≥100. Patients with a CAC score of zero are advised to reassess their score every five years until a positive CAC score is detected.

Under the 2019 ACC/AHA guidelines, without using the CAC score, patients with low-density lipoprotein cholesterol (LDL-C) levels between 70 and 189 mg/dL are prescribed a moderate-potency statin, while those with LDL-C levels of 190 mg/dL or higher are prescribed a high-potency statin.

For more clarity, we have expanded the detailed on the intervention in line 35-47 as follow: “**Objective:** This study aims to evaluate the cost-utility of Coronary Artery Calcium (CAC) screening for primary prevention in Thai patients with intermediate cardiovascular diseases (CVD) risk, compared to the current practice according to the ACC/AHA guideline 2019 recommendation without using of CAC score.

**Methods:** A hybrid model combining a decision tree and Markov model was constructed to compare cost and QALYs from a societal perspective. The model evaluated the target population of statin-naïve individuals aged 40-75 with intermediate CVD risk. We assessed the impact of statin initiation for primary prevention based on the ACC/AHA guideline 2019 with CAC screening compared to without CAC screening over a 35-year time horizon.”

References

1. Tiansuwan, N., Sasiprapha, T., Jongjirasiri, S., Unwanatham, N., Thakkinstian, A., Laothamatas, J., & Limpijankit, T. (2023). Utility of coronary artery calcium in refining 10-year ASCVD risk prediction using a Thai CV risk score. Frontiers in cardiovascular medicine, 10, 1264640. https://doi.org/10.3389/fcvm.2023.1264640

2. Stating that the old practice has a 29% of being cost-effective compared to the CAC intervention having a probability of 71% is redundant; in a comparison of two interventions the probability that the other is cost-effective is going to be 100-(probability other is cost-effective). This could be removed for space and replaced with other information.

**Answer:** We appreciated your feedback. We have removed “29% for current guideline” according to your suggestion.

3. Recommend rounding all dollar figures, including ICERs, to the nearest whole number (i.e. no decimal places). What is the Thai WTP threshold? It might be useful to provide a conversion to USD in the abstract for international readers.

**Answer:** Thank you for your suggestions. According to International Monetary Fund 2024, 1 US dollar is approximately 36 THB. We have rounded all cost values to the nearest whole number. According to the International Monetary Fund (2024), 1 USD is approximately 36 THB. To enhance generalizability, we have also included conversions to US dollars using this exchange rate in line 37-39 as follow: “The incremental cost-effectiveness ratio (ICER) was compared against the official willingness-to-pay threshold of Thailand, (160,000 THB, approximately 4,400 USD per QALYs).”

**Introduction:**
4. Perhaps provide a short definition in lay terms of dyslipidemia and atherosclerosis.

**Answer:** We appreciate your feedback. Dyslipidemia refers to an abnormal lipid levels, including fats, triglycerides, cholesterol, and phospholipids, which can be either too high or too low [1]. The buildup of fat and cholesterol in the artery walls forms plaque, eventually narrowing or blocking blood flow. This process, called atherosclerosis, is a key cause of cardiovascular disease (CVD) [2]. The presence of calcium in the coronary arteries is an indication of the presence of atherosclerotic plaque, which is caused by long-term exposing to the atheromatous process [3]. Both dyslipidemia and atherosclerosis are well established medical terms, included in the International Classification of Diseases (ICD) version 10, which is used globally in healthcare field [4]. However, the other field might not be familiar with these terms.

We have added the short description of those term in line 59-67 as follows: “Atherosclerotic cardiovascular diseases (ASCVD), caused by the gradual buildup of cholesterol plaques inside the arteries, are the leading cause of death globally, with ischemic heart disease and cerebrovascular disease being the two major sources of disability [1, 2]. Many studies have demonstrated the heavy economic burden of ASCVD, particularly in low- and middle-income countries (LMICs) compared to high-income countries (HICs) [3, 4]. Effective management of dyslipidemia, a disorder characterized by abnormal levels of lipid in any form, is crucial for lowering the incidence of ASCVD. Nevertheless, numerous LMICs encounter substantial difficulties in executing effective and comprehensive national strategies for the primary prevention of ASCVD [5, 6].”

**Reference**

1. Dixon, Dave L; Riche, Daniel M (April 21, 2021). "Dyslipidemia". Pharmacotherapy:A Pathophysiological Approach, 11e. Book authored by Joseph T. DiPiro, Gary C. Yee, L. Michael Posey, Stuart T. Haines, Thomas D. Nolin, Vicki Ellingrod. Archived from the original on 2020-08-08.
2. Mori H, Torii S, Kutyna M, Sakamoto A, Finn AV, Virmani R: Coronary artery calcification and its progression: what does it really mean?. JACC Cardiovasc Imaging. 2018, 11:127-42. 10.1016/j.jcmg.2017.10.012
3. Grech ED: Pathophysiology and investigation of coronary artery disease . BMJ. 2003, 326:1027-30.10.1136/bmj.326.7397.1027
4. World Health Organization. *International Classification of Diseases (ICD).* WHO. <https://www.who.int/standards/classifications/classification-of-diseases> [Accessed 1 October 2024.]

5. What is meant by “subclinical”

**Answer:** The term "subclinical" refers to diseases that present without clinical manifestations, or is "asymptomatic." This term is widely used in the medical field to describe a stage of chronic disease where there are no symptoms, but the disease can still be detected through laboratory tests, imaging, or other methods.

Atherosclerosis is a chronic, progressive, inflammatory disease with a long asymptomatic phase. As the disease progresses, it can lead eventually to the occurrence of acute cardiovascular events such as myocardial infarction, unstable angina pectoris and sudden cardiac death. While the disease is still in a subclinical stage, however, the presence of atherosclerosis can be identified by several methods, including coronary angiography, CT for Coronary Artery Calcium (CAC) testing [1-2].

Reference

1. Grech ED: Pathophysiology and investigation of coronary artery disease . BMJ. 2003, 326:1027-30.10.1136/bmj.326.7397.1027
2. Toth PP. Subclinical atherosclerosis: what it is, what it means and what we can do about it. Int J Clin Pract. 2008;62(8):1246-1254. doi:10.1111/j.1742-1241.2008.01804.x

6. Suggest modifying the sentence: “This study aims to evaluate the cost-effectiveness of CAC screening for primary prevention in Thai patients with intermediate ASCVD risk, compared to the current guidelines according to the ACC/AHA guidelines 2019 recommendation” to be clearer. Propose something like: “This study aims to evaluate the cost-effectiveness of including CAC screening for primary prevention in Thai patients with intermediate ASCVD risk as per the ACC/AHA guidelines 2019 recommendation, compared to the current Thai guidelines which do not advise screening in this population.”

**Answer:** We appreciate your feedback and apologize for any confusion in our manuscript. As mentioned, our goal is to compare the impact on cost-utility of using the CAC score as a cardiovascular risk assessment tool based on the ACC/AHA guideline 2019 (our index strategy) versus the ACC/AHA guideline 2019 recommendations without using CAC score (comparison strategy). We did not directly compare our model to the Thai clinical guidelines because they are likely underutilized due to being outdated and may not reflect current practices. Although there are minor differences between the 2016 Thai guidelines [1] and the ACC/AHA guideline 2019 [2] regarding statin use based on LDL levels, the use of the CAC score in Thailand was quite limited between 2016 and 2019. Awareness of CAC screening has increased significantly since the publication of the 2019 ACC/AHA guidelines [2], which provide more specific recommendations on the timing of CAC screening.

For more clarification, we have replaced the term "current guideline" with "current practice" and we have re-structed the final paragraph of introduction in line 80- 93 as follow: “Previous health economic studies have demonstrated the cost-effectiveness of CAC testing for primary prevention [16-21]. Based on the American College of Cardiology/American Heart Association (ACC/AHA) Cholesterol management guideline 2019, CAC testing for informed decisions in intermediate-risk patients appears reasonable [9]. However, the Thailand Clinical Practice Guideline on Pharmacologic Therapy of Dyslipidemia for Atherosclerotic Cardiovascular Disease (ASCVD) Prevention 2016 did not implement the supplemental use of CAC scores for risk stratification [8]. Additionally, there was a lack of local evidence to support the cost-effectiveness of CAC screening in the Thai context. Consequently, the use of CAC screening in the country is currently guided by the 2019 ACC/AHA recommendations [9]. The inclusion of CAC for ASCVD risk screening in the publicly financed health insurance scheme needs to be justified [22]. This study aims to evaluate the cost-effectiveness of using CAC screening strategy to guide statin initiation for primary prevention in Thai patients with intermediate ASCVD risk, compared to current practice based on the ACC/AHA 2019 guidelines without incorporating the CAC screening.”

And in method section in line 116-129 as follow: “A decision tree was used to compare the costs and consequences of two screening strategies: (1) CAC screening and (2) Current practice (Fig 1). For the CAC screening strategy, individual patients are assessed for their CAC score to guide statin therapy. A moderate-potency statin is initiated for patients with a CAC score of 1-99, and a high-potency statin is initiated for patients with a CAC score of ≥100. Patients with a CAC score of zero are recommended to reassess the score every 5 years until a positive CAC score is detected [6]. All simulated individuals are assumed to receive lifestyle advice.

The current practice reflects the implementation of the ACC/AHA guideline 2019 without incorporating the CAC screening [6] where statin therapy is initiated under the following conditions: for a low-density lipoprotein cholesterol (LDL-C) of 70-189 mg/dL, a moderate-potency statin is provided, and for an LDL-C of ≥190 mg/dL, a high-potency statin is provided. All patients are assumed to maintain their statin potency level without stepping down. We did not directly compare CAC screening strategy to the Thai clinical guidelines 2016 [8] because they are likely underutilized due to being outdated and may not reflect current practices.”

Reference

1. The Royal College of Physicians of Thailand., 2016. RCPT clinical practice guideline on pharmacologic therapy of dyslipidemia for atherosclerotic cardiovascular disease prevention. Available: http://www.thaiheart.org/images/column_1487762586/2016%20RCPT%20Dyslipidemia%20Clinical%20Practice%20Guide line.pdf [Accessed September 30, 2023]
2. Arnett, D. K., Blumenthal, R. S., Albert, M. A., Buroker, A. B., Goldberger, Z. D., Hahn, E. J., Himmelfarb, C. D., Khera, A., Lloyd-Jones, D., McEvoy, J. W., Michos, E. D., Miedema, M. D., Muñoz, D., Smith, S. C., Jr, Virani, S. S., Williams, K. A., Sr, Yeboah, J., & Ziaeian, B. (2019). 2019 ACC/AHA Guideline on the Primary Prevention of Cardiovascular Disease: A Report of the American College of Cardiology/American Heart Association Task Force on Clinical Practice Guidelines. Circulation, 140(11), e596–e646. https://doi.org/10.1161/CIR.0000000000000678

**Model Overview:**

7. Revise first sentence “A hybrid model combining a decision tree and a hybrid model combining a decision tree and Markov model was constructed (Fig 1 and 2)” – sounds like “hybrid model combining a decision tree” is repeated?

**Answer:** We apologize for the mistake of repeating "a hybrid model combining a decision tree" twice. We have corrected this typo based on your suggestion.

8. “Quality-adjusted life-years (QALYs) were used as the outcome utility measure.” I think you can delete “utility” and just say outcome measure.

**Answer:** We have edited according to your suggestions.

9. Can a reference be provided for the Thai guidelines?

**Answer:** Thank you for suggestion. The reference for the Thai guidelines was provided as reference number 24 as the following:

24. Health technology assessment (HTA) process guideline. Health Intervention and Technology Assessment Program (HITAP). Available from: https://www.hitap.net/wp-content/uploads/2014/09/health_technology_assessment_process_guidelines_english_finalversion_01march2012.pdf. [Accessed 1 February 2024.]

**Data Sources**
10. “Patient identity was access only during the data collection and was not collected.” Suggest “viewed only during data collection and was not recorded”

**Answer:** We have edited according to your suggestion

11. Table 1: since all have the same distribution and source, these columns could be combined/moved to a footnote of the table.

**Answer:** We have edited according to your suggestion. Table below is the edited versions

**Table 1. Selected input parameters to the decision tree model**

| **Input parameter: probabilities** | **Base-case value**  **mean (SE)** |
| --- | --- |
| **Current guideline strategy** |  |
| Incidence of initial LDL-C ≥ 190 mg/dL | 0.018 (0.013) |
| Incidence of initial LDL-C 70-189 mg/dL | 0.759 (0.04) |
| Incidence of initial LDL-C <70 mg/dL | 0.223 (0.04) |
| **CAC screening strategy** |  |
| Incidence of initial CAC ≥ 100 | 0.36 (0.013) |
| Incidence of initial CAC 1-99 | 0.39 (0.013) |
| Incidence of initial CAC = 0 | 0.26 (0.012) |

***The input data were sourced from the hospital database from 2012 to 2022. A beta distribution was selected for these input parameters in the probabilistic sensitivity analyses. Abbreviation:** CAC, coronary calcium score; LDL-C, low-density lipoprotein cholesterol; SE, standard error

12. Table 2: Personally I think five decimal places is a lot, I think displaying four in the table would be OK. As commented for the abstract rounding costs to whole number is advised. What is the utility for the “No CVD” health state?

**Answer:** We appreciate your feedback and would like to address your concern. In this field, the estimated rate of CVD development is often reported per 1,000 person-years. For example, the incidence rate of CVD is estimated at 6.25 events per 1,000 person-years. When converted to align with the cycle of our study, this becomes 0.00625 per person-year. Given that our study uses a long time horizon (35 years), reporting with more decimal places becomes significant in term of accuracy and reproducibility. Certain studies in this field [1-2] also report values with five or more decimal places.

Regarding input costs, we have made the necessary revisions based on your suggestion. The utility values for the "No CVD" health state by age are provided in Supplementary Table S4 as shown below.

| Age |  | Death (Gen pop) | Utility gen pop |
| --- | --- | --- | --- |
|  |  |  |  |
| 40 |  | 0.0038 | 0.941 |
| 41 |  | 0.0038 | 0.941 |
| 42 |  | 0.0038 | 0.941 |
| 43 |  | 0.0038 | 0.941 |
| 44 |  | 0.0038 | 0.941 |
| 45 |  | 0.0050 | 0.923 |
| 46 |  | 0.0050 | 0.923 |
| 47 |  | 0.0050 | 0.923 |
| 48 |  | 0.0050 | 0.923 |
| 49 |  | 0.0050 | 0.923 |
| 50 |  | 0.0069 | 0.923 |
| 51 |  | 0.0069 | 0.923 |
| 52 |  | 0.0069 | 0.923 |
| 53 |  | 0.0069 | 0.923 |
| 54 |  | 0.0069 | 0.923 |
| 55 |  | 0.0099 | 0.886 |
| 56 |  | 0.0099 | 0.886 |
| 57 |  | 0.0099 | 0.886 |
| 58 |  | 0.0099 | 0.886 |
| 59 |  | 0.0099 | 0.886 |
| 60 |  | 0.0141 | 0.886 |
| 61 |  | 0.0141 | 0.886 |
| 62 |  | 0.0141 | 0.886 |
| 63 |  | 0.0141 | 0.886 |
| 64 |  | 0.0141 | 0.886 |
| 65 |  | 0.0211 | 0.862 |
| 66 |  | 0.0211 | 0.862 |
| 67 |  | 0.0211 | 0.862 |
| 68 |  | 0.0211 | 0.862 |
| 69 |  | 0.0211 | 0.862 |
| 70 |  | 0.0320 | 0.862 |
| 71 |  | 0.0320 | 0.862 |
| 72 |  | 0.0320 | 0.862 |
| 73 |  | 0.0320 | 0.862 |
| 74 |  | 0.0320 | 0.862 |
| 75 |  | 0.0503 | 0.862 |

For more clarification, we have added the footnote in Table 2 as follow: “The utility of No CVD health state was provided in Supplementary Table S4”

Reference

1. Roberts ET, Horne A, Martin SS, et al. Cost-effectiveness of coronary artery calcium testing for coronary heart and cardiovascular disease risk prediction to guide statin allocation: the Multi-Ethnic Study of Atherosclerosis (MESA). PLoS One 2015;10:e0116377.
2. Galper BZ, Wang YC, Einstein AJ. Strategies for primary prevention of coronary heart disease based on risk stratification by the ACC/ AHA lipid guidelines, ATP III guidelines, coronary calcium scoring, and C-reactive protein, and a global treat-all strategy: a comparativeeffectiveness modeling study. PLoS One 2015; 10:e0138092.

**Sensitivity Analysis:**
13. What about a scenario analysis where the discount rate is varied (or set to zero)?
-“MESA” acronym – needs to be defined and described

**Answer:** Thank you for your suggestions. We have included the discount rate in the one-way sensitivity analysis, varying it between 0% and 5% for both outcomes and costs [1], as illustrated in the red box in Figure 4 below. Additionally, we have added text to address this step in lines 195-199 as follows.: “Beyond the PSA, we performed several additional sensitivity analyses to assess the impact of important assumptions. The following input parameters were evaluated for their ICER response by varying upper and lower range values and were presented using a tornado diagram: the incidence of initial LDL-C levels and CAC scores, transition probability of the first CVD event, statin price, treatment effectiveness, health state utilities, and discount rate (between 0% to 5%) [38].”

**Fig 4.** Tornado diagram of one-way sensitivity analysis

**Discount rate was varied between 0 -5%. Abbreviatio**n: CAC, cor onary calcium score; CT, computed tomography; CVD, cardiovascular disease; LDL, low-density lipoprotein; MI, myocardial infarction; RR, relative risk; ICER, incremental cost-effectiveness ratio

We apologized for not providing more detail for MESA cohort. MESA or Multu-Ethnic Study of Atherosclerosis cohort is a large, ongoing medical research study in the United States that aims to investigate the prevalence, causes, and progression of subclinical cardiovascular disease (CVD) in a diverse population [2]. MESA began in 2000 consists of over 6,800 men and women, aged 45 to 84, who were free of clinical cardiovascular disease at the time of enrollment. The participants are from four major ethnic groups: White, African American, Hispanic, Chinese American. The MESA cohort is pivotal in advancing the understanding of cardiovascular disease, especially in its early stages and across diverse populations. Its use of advanced imaging, long-term follow-up, and multi-ethnic focus has provided critical insights that have influenced clinical guidelines [3-4], improved cardiovascular risk prediction, and contributed to more personalized, effective prevention and treatment strategies. We have extended more detail on this cohort in line 216-226 as follow: “Lastly, we considered the effect of changing the incidence of initial CAC scores from Tiansuwan N.-derived incidences [13] to those of a lower-risk population. Therefore, we compared our base-case set with models that assumed incidence rates from the Multu-Ethnic Study of Atherosclerosis (MESA) cohort [11], a large, ongoing medical research study in the United States that aims to investigate the prevalence, causes, and progression of subclinical CVD in a diverse population. MESA, initiated in 2000, includes over 6,800 participants aged 45 to 84 from four major ethnic groups (White, African American, Hispanic, and Chinese American) who were free of cardiovascular disease at enrollment. The study's focus on advanced imaging, long-term follow-up, and diverse populations has significantly enhanced the understanding of early cardiovascular disease, and improved risk prediction.”

Reference

1. Markus Haacker, Timothy B Hallett, Rifat Atun, On discount rates for economic evaluations in global health, *Health Policy and Planning*, Volume 35, Issue 1, February 2020, Pages 107–114, <https://doi.org/10.1093/heapol/czz127>
2. Budoff, M. J., Young, R., Burke, G., Jeffrey Carr, J., Detrano, R. C., Folsom, A. R., Kronmal, R., Lima, J. A. C., Liu, K. J., McClelland, R. L., Michos, E., Post, W. S., Shea, S., Watson, K. E., & Wong, N. D. (2018). Ten-year association of coronary artery calcium with atherosclerotic cardiovascular disease (ASCVD) events: the multi-ethnic study of atherosclerosis (MESA). European heart journal, 39(25), 2401–2408. https://doi.org/10.1093/eurheartj/ehy217.
3. Arnett, D. K., Blumenthal, R. S., Albert, M. A., Buroker, A. B., Goldberger, Z. D., Hahn, E. J., Himmelfarb, C. D., Khera, A., Lloyd-Jones, D., McEvoy, J. W., Michos, E. D., Miedema, M. D., Muñoz, D., Smith, S. C., Jr, Virani, S. S., Williams, K. A., Sr, Yeboah, J., & Ziaeian, B. (2019). 2019 ACC/AHA Guideline on the Primary Prevention of Cardiovascular Disease: A Report of the American College of Cardiology/American Heart Association Task Force on Clinical Practice Guidelines. Circulation, 140(11), e596–e646. <https://doi.org/10.1161/CIR.0000000000000678>
4. Stone, N. J., Robinson, J. G., Lichtenstein, A. H., Bairey Merz, C. N., Blum, C. B., Eckel, R. H., Goldberg, A. C., Gordon, D., Levy, D., Lloyd-Jones, D. M., McBride, P., Schwartz, J. S., Shero, S. T., Smith, S. C., Jr, Watson, K., Wilson, P. W., & American College of Cardiology/American Heart Association Task Force on Practice Guidelines (2014). 2013 ACC/AHA guideline on the treatment of blood cholesterol to reduce atherosclerotic cardiovascular risk in adults: a report of the American College of Cardiology/American Heart Association Task Force on Practice Guidelines. Journal of the American College of Cardiology, 63(25 Pt B), 2889–2934. https://doi.org/10.1016/j.jacc.2013.11.002

**Results**

14. Table 4: suggest revising the title, it is currently not clear/grammatically correct. I don’t think stating something is “more dominant” or “less dominant” is appropriate. CAC is not dominant in the base case. I think just stating whether it is or is not dominant is appropriate.

**Discussion**

15. Using “dominant” to describe the results is not appropriate. To me this terminology is only used when an intervention is both less costly and more effective. The CAC strategy is cost-effective but not dominant. If it becomes dominant in a scenario analysis that can be stated, but stating “more dominant” or “less dominant”, does not align with my understanding of the base case findings.

**Answer (14, 15):** Thank you for your suggestions, we apologized for the misconception. There are four corresponding CEA results [1]: pharmaceutical care could be dominant (less costly and more effective) in the bottom right quadrant of cost-effectiveness plane; more effective and more costly (top right); dominated (less effective and more costly) in the top left quadrant; or less costly and less effective in the bottom left. We have made all the necessary corrections in the manuscript as follow:

**Table 4. Sensitivity analysis based on scenario**

| **Scenario** | |  | **Incremental**  **Cost (THB)** | **Incremental QALYs (years)** | **ICER** | **Decision*** |
| --- | --- | --- | --- | --- | --- | --- |
| **Drug adherence** | | | | | |  |
| Current practice | | CAC screening |  |  |  |  |
| 19% | | 36% for CAC =0  52% for CAC 1-99  56% for CAC ≥ 100 | -69,3670 | 1.07 | -65,090 | CAC is dominant |
| 30% | |  | -45,636 | 0.88 | -51,834 | CAC is dominant |
| 52% | |  | 5,106 | 0.49 | 10,433 | CAC is cost-effective |
| 100% (base case) | | 100% (base case) | 10,091 | 0.62 | 16,308 | CAC is cost-effective |
|  | |  |  |  |  |  |
| **Cost parameter (THB)** | | | | | |  |
| High potency statin | | CAC test |  |  |  |  |
| 5,479 (15 per unit) | | 4,000 (base case) | -11,901 | 0.62 | -19,235 | CAC is dominant |
| 7,305 (20 per unit) | | 4,000 (base case) | -722 | 0.62 | -1,167 | CAC is dominant |
| 9,071 (25 base case) | | 4,000 (base case) | 10,091 | 0.62 | 16,308 | CAC is cost-effective |
| 10,956 (30 per unit) | | 4,000 (base case) | 21,636 | 0.62 | 34,968 | CAC is cost-effective |
| 12784 (35 per unit) | | 4,000 (base case) | 32,816 | 0.62 | 53,036 | CAC is cost-effective |
| 23,596 (64 per unit) | | 4,000 (base case) | 98,999 | 0.62 | 160,000 | CAC is not cost-effective |
|  | |  |  | 0.62 |  |  |
| 9,071 (base-case) | | 2,000 | 8,071 | 0.62 | 13,044 | CAC is dominant |
| 9,071 (base-case) | | 4,000 (base case) | 10,091 | 0.62 | 16,308 | CAC is cost-effective |
| 9,071 (base-case) | | 6,000 | 12,111 | 0.62 | 19,573 | CAC is cost-effective |
| 9,071 (base-case) | | 8,000 | 14,131 | 0.62 | 22,838 | CAC is cost-effective |
| 9,071 (base-case) | | 92,028 | 98,999 | 0.62 | 160,000 | CAC is not cost-effective |
|  | |  |  |  |  |  |
| **Incident of CVD event in patient with CAC score of 0** | | | | | |  |
| Incidence of non-fatal MI (Person-year) | | | | | |  |
| 0.00160 | |  | 8,883 | 0.65 | 13,699 | CAC is cost-effective |
| 0.00325 (base-case) | |  | 10,091 | 0.62 | 16,308 | CAC is cost-effective |
| Incidence of non-fatal stroke (Person-year) | | | | | |  |
| 0.00440 | |  | -443 | 0.70 | -634 | CAC is dominant |
| 0.00883 (base-case) | |  | 10,091 | 0.62 | 16,308 | CAC is cost-effective |
| Incidence of non-fatal CVD death (Person-year) | | | | | |  |
| 0.00052 | |  | 10,340 | 0.64 | 16,284 | CAC is cost-effective |
| 0.00104 (base-case) | |  | 10,091 | 0.62 | 16,308 | CAC is cost-effective |
|  | |  |  |  |  |  |
| **Incident of initial CAC score (Probability)** | | | | | |  |
| MESA cohort [11] | |  |  |  |  |  |
| CAC ≥ 100 | 0.24 |  | -4902 | 0.48 | -10,136 | CAC is dominant |
| CAC 1-99 | 0.26 |  |  |  |  |  |
| CAC = 0 | 0.50 |  |  |  |  |  |
| Tiansuwan, N. (base-case) [7] | | |  |  |  |  |
| CAC ≥ 100 | 0.36 |  | 10,091 | 0.62 | 16,308 | CAC is cost-effective |
| CAC 1-99 | 0.39 |  |  |  |  |  |
| CAC = 0 | 0.26 |  |  |  |  |  |
|  |  |  |  |  |  |  |
| **CAC progression parameter** | | | | | | |
| Absence | | | -6,866 | 0.62 | -11,014 | CAC is dominant |
| Based on CAC Consortium [45] | | | 13 | 0.64 | 20 | CAC is cost-effective |
| Based on Hospital data | |  | 10,091 | 0.62 | 16,308 | CAC is cost-effective |
|  | |  |  |  |  |  |

*Cost-effectiveness was interpreted under the Thai willingness-to-pay threshold of 160,000 THB per QALY gained. Approximately 36 THB = 1 US$ in 2024. **Abbreviation:** CAC, coronary calcium score; ICER, incremental cost-effectiveness ratio; QALYs, Quality adjusted life years.

**In the discussion section In line 286-289 : “**Moderate potency statins appear to be predominated potency in clinical practice according to ACC/AHA guideline 2019 [9, 40]. We found that when the annual cost of high-potency statins increased, CAC screening was more costly.”

**in line 338-341:**

“We attempted to address this through scenario analysis, assuming varying levels of drug adherence among patients. In a scenario when there was higher adherence rate in the CAC screening, the strategy became dominant and less costly than base-case.”

**in line 349-352:**

“In our scenario analysis, which assumed a 50% reduction in CVD events from the base-case value for patients with a CAC score of zero, the CAC screening strategy demonstrated a reduction in incremental cost, making it dominant strategy.”

**Reference**

1. Neumann, P. J., Ganiats, T. G., Russell, L. B., Sanders, G. D., & Siegel, J. E. (2016). Cost-effectiveness in health and medicine (2nd ed.). Oxford University Press. https://doi.org/10.1093/acprof:oso/9780190492939.001.0001

16. It's interesting to me that the cost of the statins seems to be driving the higher cost of the CAC strategy. The way the discussion phrases it, it doesn’t seem like there is a benefit to the higher dose of statins, that it is simply an extra cost to be rationalized but doesn’t lead to avoidance of downstream costs or increase in utility. Is this the intention of the discussion section on statin costs?

**Answer:** We appreciate your feedback and would like to address your concern. ASCVD is a disease with long subclinical stage. Preventing a major cardiovascular event (such as myocardial infarction, ischemic stroke, or CVD) requires long-term statin therapy. Once a patient experiences a major cardiovascular event, high-potency statins are prescribed to prevent secondary (recurrent) events. Accurately assessing cardiovascular risk and identifying patients who need long-term intensive statin therapy, or those who may not benefit, is crucial in reducing costs and effectively improving QALYs.

According to the Tornado diagram, while the effectiveness of high- and moderate-potency statins influences the cost-effectiveness in our model, their impact is much smaller compared to the cost of high-potency statins. The price of statins and how they are distributed between the two strategies therefore played a significant role. In current practice, 76% of patients are placed on moderate-potency statins, compared to approximately 30% in the CAC screening strategy. As a result, the cost of moderate-potency statins has a significantly greater impact on the overall cost in current practice than in the CAC screening approach. Increases in moderate-potency statin costs lead to relatively lower total costs for CAC screening, making it more cost-effective. Increasing of high-potency statins also lead to relatively higher total costs for CAC screening in the same way.

17. Is there any research on cost-effectiveness of CAC screening from other settings where this has already been included in guidelines?

**Answer:** According to the ACC/AHA guideline 2019, CAC screening is already recommended as an additional tool for risk stratification in patients with intermediate cardiovascular risk. Previous studies, all conducted in the USA, have demonstrated that CAC screening is cost-effective [1-5].

Reference

1. van Kempen, B. J., Spronk, S., Koller, M. T., Elias-Smale, S. E., Fleischmann, K. E., Ikram, M. A., Krestin, G. P., Hofman, A., Witteman, J. C., & Hunink, M. G. (2011). Comparative effectiveness and cost-effectiveness of computed tomography screening for coronary artery calcium in asymptomatic individuals. Journal of the American College of Cardiology, 58(16), 1690–1701. https://doi.org/10.1016/j.jacc.2011.05.056
2. Pletcher, M. J., Pignone, M., Earnshaw, S., McDade, C., Phillips, K. A., Auer, R., Zablotska, L., & Greenland, P. (2014). Using the coronary artery calcium score to guide statin therapy: a cost-effectiveness analysis. Circulation. Cardiovascular quality and outcomes, 7(2), 276–284. https://doi.org/10.1161/CIRCOUTCOMES.113.000799
3. Roberts, E. T., Horne, A., Martin, S. S., Blaha, M. J., Blankstein, R., Budoff, M. J., Sibley, C., Polak, J. F., Frick, K. D., Blumenthal, R. S., & Nasir, K. (2015). Cost-effectiveness of coronary artery calcium testing for coronary heart and cardiovascular disease risk prediction to guide statin allocation: the Multi-Ethnic Study of Atherosclerosis (MESA). PloS one, 10(3), e0116377. https://doi.org/10.1371/journal.pone.0116377
4. van Kempen, B. J., Ferket, B. S., Steyerberg, E. W., Max, W., Myriam Hunink, M. G., & Fleischmann, K. E. (2016). Comparing the cost-effectiveness of four novel risk markers for screening asymptomatic individuals to prevent cardiovascular disease (CVD) in the US population. International journal of cardiology, 203, 422–431. https://doi.org/10.1016/j.ijcard.2015.10.171
5. Hong, J. C., Blankstein, R., Shaw, L. J., Padula, W. V., Arrieta, A., Fialkow, J. A., Blumenthal, R. S., Blaha, M. J., Krumholz, H. M., & Nasir, K. (2017). Implications of Coronary Artery Calcium Testing for Treatment Decisions Among Statin Candidates According to the ACC/AHA Cholesterol Management Guidelines: A Cost-Effectiveness Analysis. JACC. Cardiovascular imaging, 10(8), 938–952. <https://doi.org/10.1016/j.jcmg.2017.04.014>

**Other comments**
18. Figure 4 needs abbreviations added. Also, the maximum and minimum ICER bars all being to one side of the base case does not make sense to me. How can this be correct? I’ve never seen a tornado diagram do that.

**Answer:** Thank you for your suggestions. The issue occurred because we initially set the vertical axis at 0. We have revised Figure 4 by adjusting the vertical axis to reflect the ICER from the base-case scenario as illustrated below.

**Fig 4.** Tornado diagram of one-way sensitivity analysis

**Discount rate was varied between 0 -5%. Abbreviatio**n: CAC, cor onary calcium score; CT, computed tomography; CVD, cardiovascular disease; LDL, low-density lipoprotein; MI, myocardial infarction; RR, relative risk; ICER, incremental cost-effectiveness ratio
