# Supplementary material for: Cost-utility analysis of Coronary Artery Calcium screening to guide statin prescription among intermediate-risk patients in Thailand
Source: PLoS One. 2025 Aug 21;20(8):e0330425. doi: 10.1371/journal.pone.0330425 (PMC12370023; doi:10.1371/journal.pone.0330425)
Supplement: Attachment — Submitted filename: Response to reviewer.docx [file pone.0330425.s005.docx]

**Reviewers comment on the manuscript #2**

**Entitles: “Cost-utility analysis of Coronary Artery Calcium screening to guide statin prescription among intermediate risk patient in Thailand”**

Dear Editor and reviewers,

We would like to thank you for your valuable reviews and comments. It is our great pleasure to have an opportunity to revise our manuscript. We have revised and modified our manuscript with some additional information (track change) as suggested by reviewers’ comment. We hope that our revisions will improve the quality of the manuscript and give a clearer vision of research methodology to meet qualification for publication in PLOS ONE. Please inform us if further information or clarification needs to be addressed. We are looking forward to your reviews and would be extremely grateful to make our response.

**Reviewer #2**

There are numerous instances of incorrect grammar, it would be an enormous effort to correct them all as a reviewer. I note that the journal does not copyedit submissions but this should be done before proceeding with publication.

**Response:** Thank you for your comments. We have carefully reviewed the manuscript and corrected all grammatical errors as suggested.

**1. Abstract: Should read “$160,00 BAHT per QALY” not QALYs**

**Answer:** Thank you for your suggestions. We have made a necessary edit throughout the manuscript.

**2. The statement: “Probabilistic, and additional one-way sensitivity analyses were performed to account for the model’s important assumption and robustness” is not clear. Suggest revising to say it is assessing robustness and testing the impact of assumptions.**

**Answer:** Thank you for your suggestions. We have edited according to your suggestion in line 46-49 as follows: “Probabilistic and one-way sensitivity analyses were conducted to assess the robustness of the model and evaluate how variations in key assumptions impact the results. These analyses help determine the reliability of the findings by exploring the extent to which changes in input parameters influence the overall conclusions.”

**3. Suggest rounding all monetary figures to the nearest whole value (i.e. remove decimals) throughout text.**

**Answer:** Thank you for your suggestions. We have made a necessary edit throughout the manuscript.

**4. Introduction line 69 and Methods line 101: what are the Thai risk assessment tools? They are not described in detail so it is hard to know what they involve.**

**Answer:** We appreciated your feedback. Thai risk assessment tool is called Thai CV risk tools [1]. The tool predicted 10-year risk of having cardiovascular event as percentage.

For more clarify, we have added more detail of the tools in line 68-72 as follows: “In subclinical individuals, primary prevention of ASCVD is based on the predicted 10-year risk of a CVD event. In Thailand, patients are assessed using Thai cardiovascular (CV) risk tools, which estimate their 10-year risk as a percentage [7]. Statin therapy is not recommended for individuals at low risk (<10% 10-year risk), while it is recommended for those at high ASCVD risk (>20% 10-year risk) [8].”

**Reference**

1. Vathesatogkit, P., Woodward, M., Tanomsup, S., Ratanachaiwong, W., Vanavanan, S., Yamwong, S., & Sritara, P. (2012). Cohort profile: the electricity generating authority of Thailand study. International journal of epidemiology, 41(2), 359–365. https://doi.org/10.1093/ije/dyq218

**5. Introduction line 79: Can a brief description of what CAC involves and what it assesses be provided?**

**Answer:** Thank you for your suggestions. We have added the short description of CAC in line 80-84 as follows: “The Coronary Artery Calcium (CAC) score, introduced in the late 1990s, is a non-invasive imaging technique used to assess coronary artery calcification [14]. It quantifies calcified atherosclerotic plaques within the coronary arteries and has been strongly associated with CVD events [15, 16]. Extensive evidence strongly supports the potential of Coronary Artery Calcium (CAC) scores as an accurate tool for cardiovascular risk stratification [17, 18].”

**6. Methods line 116: I think the terminal nodes for the different strategies in the decision trees should be described. My understanding is that after screening, a patient can be on no statin, moderate potency statin, or high potency statin. This is clear in the figure but not described in the text. I think Table 1 could also be revised with a column added to show how these classifications relate to no/moderate/high potency statins. This will make it more clear for the reader.**

**Answer:** Thank you for your suggestions. To ensure greater clarity and comprehensibility, we have added the corresponding treatment column for each terminal node in decision tree within Table 1, as presented below.

**Table 1. Selected input parameters to the decision tree model and corresponding treatment strategy**

| **Input parameter: probabilities** | **Base-case value**  **mean (SE)** | **Receiving treatment** |
| --- | --- | --- |
| **Current practice strategy** |  |  |
| Incidence of initial LDL-C ≥ 190 mg/dL | 0.018 (0.013) | High potency statin |
| Incidence of initial LDL-C 70-189 mg/dL | 0.759 (0.04) | Moderate potency statin |
| Incidence of initial LDL-C <70 mg/dL | 0.223 (0.04) | No statin |
| **CAC screening strategy** |  |  |
| Incidence of initial CAC ≥ 100 | 0.36 (0.013) | High potency statin |
| Incidence of initial CAC 1-99 | 0.39 (0.013) | Moderate potency statin |
| Incidence of initial CAC = 0 | 0.26 (0.012) | No statin |

***The input data were sourced from the hospital database from 2012 to 2022. A beta distribution was selected for these input parameters in the probabilistic sensitivity analyses. Abbreviation:** CAC, coronary calcium score; LDL-C, low-density lipoprotein cholesterol; SE, standard error

**7. Discussion Line 288: “Based on our analysis, approximately one-third of patients who followed the CAC screening strategy received moderate- or high-potency statins at the beginning, while the majority of patients in the current practice received moderate-potency statins (76%).“ As discussed in the methods feedback, would it be possible to show how many patients are on the different therapies after the decision tree portion of the model? It would make the differences in the treatment of the two groups more obvious and understandable.**

**Answer:** Thank you for your suggestions. We have added the corresponding treatment column for each terminal node in decision tree within Table 1, as presented below.

**Table 1. Selected input parameters to the decision tree model and corresponding treatment strategy**

| **Input parameter: probabilities** | **Base-case value**  **mean (SE)** | **Receiving treatment** |
| --- | --- | --- |
| **Current practice strategy** |  |  |
| Incidence of initial LDL-C ≥ 190 mg/dL | 0.018 (0.013) | High potency statin |
| Incidence of initial LDL-C 70-189 mg/dL | 0.759 (0.04) | Moderate potency statin |
| Incidence of initial LDL-C <70 mg/dL | 0.223 (0.04) | No statin |
| **CAC screening strategy** |  |  |
| Incidence of initial CAC ≥ 100 | 0.36 (0.013) | High potency statin |
| Incidence of initial CAC 1-99 | 0.39 (0.013) | Moderate potency statin |
| Incidence of initial CAC = 0 | 0.26 (0.012) | No statin |

***The input data were sourced from the hospital database from 2012 to 2022. A beta distribution was selected for these input parameters in the probabilistic sensitivity analyses. Abbreviation:** CAC, coronary calcium score; LDL-C, low-density lipoprotein cholesterol; SE, standard error

**8. Methods: Can more details be provided about who the cardiologists and health economists were who reviewed the model, and what the review process involved?**

**Answer**: The conceptualisation and initial drafting of the model structure were carried out with the valuable contributions of cardiologist Pannipa Suwannasom. The study’s model was then reviewed and validated by external cardiologists, including Songsak Kiatchoosakun, Suphot Srimahachota, Arintaya Phrommintikul, and Yotsawee Chotechuang. Furthermore, the economic aspects of the model were thoroughly reviewed by Surasak Saokaew. The process involved multiple meetings, beginning with designing the model structure and validating the data used for input parameters. Follow-up meetings were then conducted to review and discuss both the preliminary and final results.

We have acknowledged the external experts who assisted in this study in the Acknowledgements section, line 378 - 382, as follows: “This study was partially supported by the Faculty of Medicine, Chiang Mai University, the University of Phayao, and the Thailand Science Research and Innovation Fund. We extend our deep gratitude to cardiologists Songsak Kiatchoosakun, Suphot Srimahachota, Arintaya Phrommintikul, and Yotsawee Chotechuang for their valuable review and validation of the model.”

**9. Table formatting looks off in my version. Will need to be revised to common font and layout in publication.**

**Answer:** We appreciate your feedback. The font in all tables has been revised accordingly.

**10. Table 2: What was cost of CAC testing? I don’t think this is shown in the costs section?**

**Answer:** We appreciate your feedback and sincerely apologise for any confusion in our manuscript. The cost of CAC testing is provided in Table 2. However, we initially used the term "CT coronary calcium scan," which may not be familiar to experts outside the field. To enhance clarity and consistency, we have revised it to "CAC screening" throughout the manuscript.

**11. Table 2: Perhaps put non-medical costs in their own category so they are more obvious. Will also need to describe how these were gathered. Is it from a patient survey? There is very little detail on how these costs were obtained. Perhaps conducting a scenario analysis from the health system perspective would also be of interest.**

**Answer:** We appreciate your feedback and would like to address your concern regarding the non-medical costs associated with non-fatal MI. These costs were sourced from Anukoolsawat et al. (2006) [1], where interview records from 193 ACS patients were used to estimate direct non-medical expenses. The non-fatal stroke costs was based on data from Rattanavipapong et al. (2022) [2]. This study utilized retrospective patient data from a multicentre stroke unit, incorporating key parameters such as inpatient length of stay, number of follow-up visits, and travel distance to estimate non-medical costs. Furthermore, transportation, caregiver, food, and accommodation expenses incurred during treatment were referenced from Singhpoo K et al. (2009) [3]. To enhance transparency, we have added these details into the supplementary data on page 8-9, as outlined below.: “**Details of Cost Parameters from Anukoolsawat et al. (2006) and Rattanavipapong et.al. (2022)**

Our study derived costs for secondary treatment and prevention in populations who experienced non-fatal MI costs, fatal MI costs, and direct non-medical costs from Anukoolsawat et al. (2006) [9]. This study used data from the Thai Acute Coronary Syndrome (ACS) registry to estimate lifetime costs of ACS. Medical records of 330 ACS patients were used to calculate direct medical costs (pre-event and MI-related costs of non-fatal MI), while interview records of 193 ACS patients were used to estimate direct non-medical costs. For the costs of non-fatal stroke and recurrent non-fatal stroke (both direct and indirect), our study utilized data from Rattanavipapong et al. (2022) [8] due to its recency and comprehensive. This study utilized retrospective patient data from a multicentre stroke unit, incorporating key parameters such as inpatient length of stay, number of follow-up visits, and travel distance to estimate non-medical costs. Furthermore, transportation, caregiver, food, and accommodation expenses incurred during treatment were referenced from Singhpoo K et al. (2009) [11]. Detailed information on direct and non-medical costs and their components can be found in the supplementary materials of the original article.”

We performed sensitivity analysis using health care providers perspective. The incremental cost, QALYs, and ICER for CAC screening are presented in the table below. CAC screening remains cost-effective under Thailand’s willingness-to-pay threshold of 160,000 THB per QALY gained.

| **Scenario** | | **Incremental**  **Cost (THB)** | **Incremental QALYs (years)** | **ICER** | **Decision*** |
| --- | --- | --- | --- | --- | --- |
| **Perspective** | |  |  |  |  |
| Healthcare provider perspective | | 24,762 | 0.62 | 40,020 | CAC is cost-effective |
| Societal perspective (base-case) | | 10,091 | 0.62 | 16,308 | CAC is cost-effective |
|  |  |  |  |  |  |

We have added the relevant text addressing this issue in the Methods section, line 236-237, as follows: “The post-hoc sensitivity analysis using the healthcare provider’s perspective was performed to account for the impact of direct non-medical costs.”

And in result section, line 286-288 as follows: “The post-hoc sensitivity analysis using the healthcare provider’s perspective demonstrated that CAC screening remains a cost-effective strategy (Table 4).”

1. Anukoolsawat, Pongchai, Piyamitr Sritara and Yot Teerawattananon. “Costs of Lifetime Treatment of Acute Coronary Syndrome at Ramathibodi Hospital.” (2006).
2. Rattanavipapong W, Worakijthamrongchai T, Soboon B, et al. Economic evaluation of endovascular treatment for acute ischaemic stroke in Thailand. BMJ Open2022;12:e064403. doi:10.1136/bmjopen-2022-064403
3. Singhpoo K, Tiamkao S, Ariyanuchitkul S, Sangpongsanon S, Kamsa-ard S, Lekbunyasin O, Soommart Y. The expenditures of stroke outpatients at Srinagarind hospital. Srinagarind Medical Journal. 2009;24(1):54-9.

**12. Table 2: Relative risk compared to placebo – I think placebo is what it was compared to in the source trials, but here shouldn’t it be compared to “no statin”?**

**Answer:** Thank you for your suggestions. We have edited according to your suggestions.

**13. Table 2 and methods: reference 31 used for costs is from 2006. How were these costs inflated to present values? What year is the currency presented in? This is a requested item in the CHEERS list. Perhaps add a completed CHEERS checklist as an appendix.**

**Answer:** We appreciate your feedback and would like to address your concern. All Cost data was converted to 2024 values using the Thai consumer price index (CPI) [1] and are expressed in Thai Baht (THB) (approximately 36 THB = 1 US$ in 2024) [2]. To account for time preference in the model, future costs and QALYs beyond the first year were discounted by 3% annually. This information has been explicitly stated in method section, line 185-188 as follows: “Cost data were converted to 2024 values using the Thai consumer price index (CPI) [39] and presented in Thai Baht (THB) (approximately 36 THB = 1 US$ in 2024) [40]. Future costs and QALYs beyond the first year were discounted by 3% annually.” According to your suggestion, we have added the CHEERS checklist as a supplementary file.

Reference

1. Bureau of Trade & Economics Indices Ministry of Commerce. Consumer price index of Thailand year 2024 base year 2006, 2018, 2021, and 2023. Available: http://www.price.moc.go.th/price/cpi/index_new_all.asp [Accessed 8 January 2024].
2. International Monetary Fund. World Economic Outlook Database for October 2007. Available from: https://www.imf.org/external/np/fin/ert/GUI/Pages/Report.aspx [Accessed 10 May , 2024].
